# Cushioned Insoles for First Metatarsophalangeal Joint Osteoarthritis: Protocol for the SOLE Randomised Controlled Trial

**DOI:** 10.1002/jfa2.70185

**Published:** 2026-07-18

**Authors:** Kade L. Paterson, Kim L. Bennell, Adam Bryant, Peixuan Li, Anurika P. De Silva, Sam Shearer, Rana S. Hinman

**Affiliations:** ^1^ Centre for Health, Exercise and Sports Medicine, Department of Physiotherapy, School of Health Sciences, Faculty of Medicine Dentistry & Health Sciences The University of Melbourne Melbourne Australia; ^2^ Centre for Epidemiology and Biostatistics, Melbourne School of Population and Global Health The University of Melbourne Melbourne Australia; ^3^ Methods and Implementation Support for Clinical and Health (MISCH) Research Hub, Faculty of Medicine, Dentistry and Health Sciences The University of Melbourne Melbourne Australia

**Keywords:** biomechanics, clinical trial, cushioning, first metatarsophalangeal, foot, insoles, osteoarthritis, pain, RCT

## Abstract

**Introduction:**

First metatarsophalangeal (MTP) joint osteoarthritis (OA) is a common and disabling condition that causes substantial pain and impairs quality of life. Cushioned insoles have the potential to lower plantar pressures beneath the hallux and possibly reduce pain, but their clinical effectiveness has not been investigated. This study describes the protocol for a randomised controlled trial (RCT) assessing whether daily use of cushioned insoles produces greater reductions in first MTP joint walking pain, and other symptoms, over 12 weeks than sham insoles.

**Methods and Analysis:**

This two‐arm, parallel group, superiority RCT will recruit 108 community‐dwelling adults aged 45 years and older with symptomatic radiographic first MTP joint OA. Participants will be randomised to receive three pairs of either cushioned insoles (6 mm low‐density ethyl vinyl acetate (EVA)) or visually similar thin sham insoles (2 mm high‐density EVA), to be worn for 12 weeks. The primary outcome is the 12‐week change in first MTP joint pain during walking, assessed using an 11‐point numerical rating scale (0–10, higher scores worse pain). Secondary outcomes include 12‐week changes in physical function, other measures of first MTP joint pain, health‐related quality of life, physical activity, and fear of movement. Global ratings of change in pain and function will also be assessed at 12 weeks. Other measures will also be collected. Analyses will include all participants as randomised using linear and log‐binomial regression adjusted for baseline values. Prespecified sensitivity and moderator analyses will also be conducted.

**Ethics and Dissemination:**

This study has been approved by the University of Melbourne Greater than Low Risk Human Research Ethics Committee. Findings will provide the first evidence regarding the efficacy of cushioned insoles for the management of first metatarsophalangeal joint OA. Results will be disseminated via peer‐reviewed journals, scientific conferences, information on our website and lay summaries for participants.

**Trial Registration:**

Prospectively registered on the 14 of December 2023 with the Australian New Zealand Clinical Trials Registry, reference ACTRN12623001304628

**Abbreviations**
BMI: Body mass index
FHSQ: Foot Health Status Questionnaire
FPI: Foot Posture Index
MCID: Minimal clinical important difference
NRS: Numerical rating scale
PLS: Plain Language Statement
RCT: Randomised clinical trial
SPIRIT: Standard Protocol Items Recommendations for Intervention Trials

## 1 Introduction

First metatarsophalangeal (MTP) joint osteoarthritis (OA) is a common and disabling condition [1]. Almost 1 in 10 people aged over 50 years have first MTP joint OA, with almost three quarters reporting a disabling level of pain [1]. The condition causes joint structural changes, such as joint space narrowing and dorsal osteophyte formation, which collectively result in reduced dorsiflexion range of motion of the hallux [2, 3]. This causes altered gait mechanics that increase plantar loading beneath the first ray [4], and leads to pain, impaired walking ability and reduced quality of life [2].

Clinical practice guidelines for OA advocate non‐surgical, non‐pharmacological management as first‐line care [5, 6, 7, 8]. However, randomised controlled trial (RCT) evidence to guide management for first MTP joint OA is sparse. With limited evidence specifically for first MTP joint OA, findings from trials in people with knee and hip OA are often extrapolated to the foot, even though interventions that are effective for one joint may not necessarily be effective for another. For example, we have shown that stable supportive shoes are superior to flat flexible shoes for managing knee OA pain [9], but not for pain caused by hip OA [10]. The few trials evaluating non‐surgical non‐pharmacological treatments for first MTP OA have produced mixed, or clinically uncertain, findings. For example, trials have reported within‐group pain reductions that exceeded the minimal clinically important difference (MCID) in pain for both rocker‐sole shoes and prefabricated foot orthoses [11], and for contoured foot orthoses and sham flat insoles [12], however there were no between‐group differences for either trial. Another study found a statistically significant between‐group reduction in pain with shoe‐stiffening inserts versus a sham insole, however the magnitude of improvement was below the MCID, suggesting the therapeutic effect is uncertain [13].

People with first MTP joint OA have elevated peak [4, 14] and mean [15] plantar pressures beneath the hallux, and a greater plantar pressure loading rate [14]. It is possible that these elevated pressures increase forces within and around the first MTP joint, leading to increased pain. Reducing these pressures may therefore be a plausible approach to reducing symptoms. Although one study found no relationship between reductions in plantar pressure with contoured foot orthoses and rocker‐soled shoes and perception of overall improvement (measured using a 15‐point Likert scale) [16], the study was limited in that it did not explore linear associations between changes in plantar pressure and pain severity. Thus, it remains plausible that reducing plantar pressures may improve pain in people with first MTP joint OA.

Cushioned insoles have been shown to reduce plantar pressures, with softer, thicker foams yielding greater reductions than firmer, thinner foams [17]. No trial has evaluated the effects of cushioned insoles for the management of first MTP joint OA pain. If proven effective, this would provide a simple, low burden and relatively inexpensive treatment option for this neglected condition. This study describes the protocol for an RCT that aims to assess whether daily use of cushioned insoles produce greater improvements in first MTP joint pain during walking than sham (thin) insoles over 12 weeks in people with symptomatic radiographic first MTP joint OA. Secondary aims are to evaluate the effects of cushioned insoles on other pain measures, foot‐specific function, global improvement in pain and function, health‐related quality of life, physical activity and fear of movement at 12 weeks.

## 2 Methods and Analysis

### 2.1 Study Design

The cushioned inSOLEs for first metatarsophalangeal joint osteoarthritis (SOLE) trial is a two‐arm, parallel group, superiority RCT, conducted at the Centre for Health, Exercise and Sports Medicine (CHESM), University of Melbourne. This protocol for the SOLE trial is described according to the Standard Protocol Items: Recommendations for Intervention Trials (SPIRIT; see Supporting Information S1: Table S1) [18]. The trial was prospectively registered on the 14th of December 2023 with the Australian New Zealand Clinical Trials Registry (ACTRN12623001304628). Recruitment began in February 2024 and is expected to be completed by November 2026. Reporting will be in accordance with TIDieR checklist [19] and CONSORT guidelines [20].

### 2.2 Participants

Participants are recruited from the community of greater Melbourne and regional Victoria, Australia, using advertisements in print and social media. Participants are eligible if they meet the following inclusion criteria, based on our previous RCT in first MTP OA [21] and consistent with clinical diagnostic criteria for OA [8]:

- Aged 45 years or older;
- First MTP joint pain on most days for the past 12 weeks;
- Average first MTP joint pain during walking ≥ 4 on an 11‐point numerical rating scale (NRS) (0 = no pain, 10 = worst pain imaginable) over the previous week;
- Radiographic evidence of first MTP joint OA (osteophyte or joint space narrowing score ≥ 2 on a validated atlas [22]) on weight‐bearing dorsoplantar and lateral x‐rays; and
- No morning stiffness in the first MTP joint, or morning stiffness that lasts less than 30 min.

Participants are not eligible if they exhibit:

- Prior first MTP joint surgery or planned foot surgery on the affected side in the next 12 weeks;
- Current use of foot orthoses, custom footwear or ankle braces;
- Regular primary use of footwear that prevents insole wear (e.g., high heels, flip flops/thongs);
- First MTP joint injection in the past 6 months or planned injection in the next 12 weeks;
- Other muscular, joint or neurological condition(s) rendering the participant housebound or non‐ambulant;
- Pain elsewhere in the foot/ankle that is worse than the pain in the study first MTP joint;
- Self‐reported systemic inflammatory joint disease (e.g., rheumatoid arthritis, gout);
- Moderate–severe hallux valgus (hallux abductus angle > 20°) on x‐ray [23];
- Current or planned use of a gait aid or concurrent treatment (e.g., weight loss or exercise) for first MTP joint OA in the next 12 weeks;
- Inability to understand written/spoken English or to comply with study procedures; or
- Current participation in another OA clinical trial.

Although interventions are applied bilaterally, the most symptomatic foot is deemed the study foot with respect to outcome measurement for participants with bilaterally eligible feet.

### 2.3 Procedure

**FIGURE 1:**
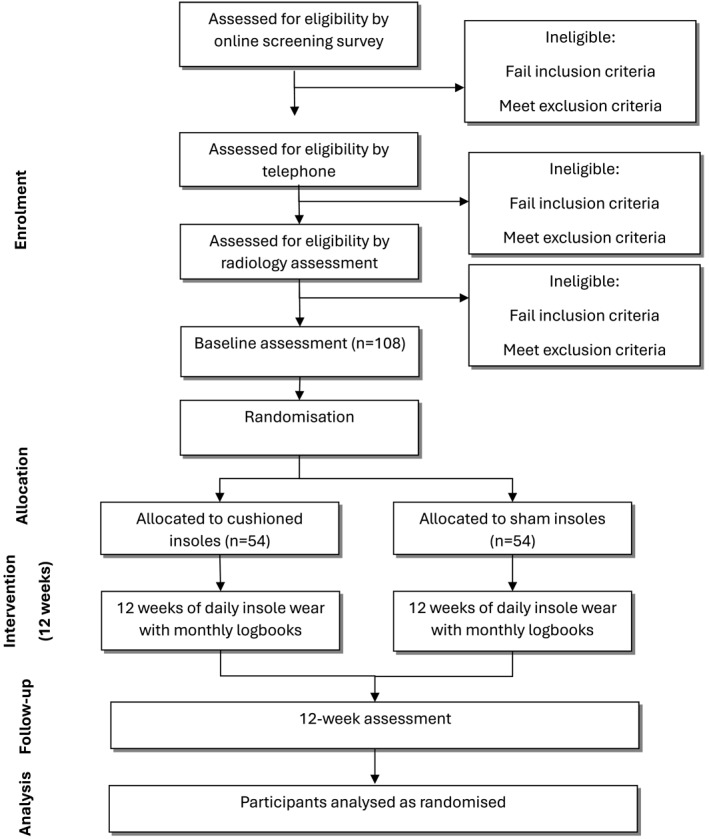
Participant flow through the study.

The flow of participants through the study is shown in Figure 1. Participants are initially screened via an online form and then via telephone interview with the Trial Coordinator. If they pass initial screening, they are referred for standardised dorsoplantar and lateral weight‐bearing x‐rays, or if they have had a suitable x‐ray in the previous 12 months, they are asked to provide these to the Trial Coordinator. X‐rays are assessed by the Principal Investigator to confirm eligibility.

**FIGURE 2:**
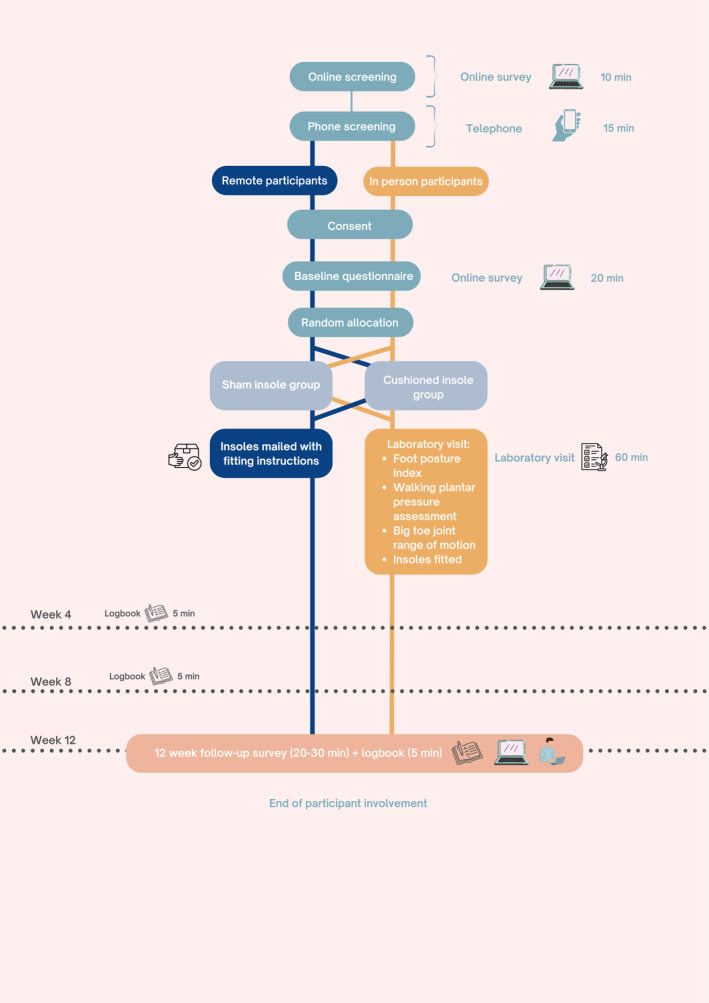
Participant experience of study processes.

Eligible participants are then provided with written and verbal information regarding the study, and are required to provide informed consent electronically via REDCap software [24]. If they consent to participate and can attend in person, they are booked to attend the Human Movement Laboratory, at the University of Melbourne. Baseline assessments are completed electronically in the laboratory on a tablet computer using REDCap software. Participants who are unable to attend the University of Melbourne are emailed a link to complete the survey at home using REDCap. After completing baseline assessments, participants are enroled in the study and randomised to receive either cushioned or sham insoles (see ‘Interventions’ below). At the 12‐week follow up, participants complete the same questionnaire as baseline electronically at home. The participant's experience of study processes is outlined in Figure 2.

### 2.4 Randomisation and Blinding

Eligible, consenting participants are randomised in a 1:1 ratio to the cushioned insoles group or the sham insoles group using randomly permuted blocks of varying sizes. The allocation sequence is generated by an independent biostatistician, stored in the REDCap database, and maintained by a researcher not involved in participant recruitment or outcome assessment. To preserve blinding of participants to the alternate group, the consent process uses limited disclosure. Participants are informed that two different types of insoles are being compared but are not told the study hypotheses nor which features of the insole the study is testing, consistent with limited disclosure processes in our prior trials [25, 26]. As participants are blinded, and are the assessors of the self‐reported outcomes, the trial is considered assessor‐blinded. An unblinded staff member records baseline descriptive characteristics, allocates participants to insole group, and fits the insoles. Participants unable to attend the laboratory self‐report their descriptive characteristics and fit the insoles themselves (see ‘Interventions’ below). The Statistical Analysis Plan will be written while the biostatisticians are blinded to group allocation and published on our research centre's website prior to unblinding and statistical analysis.

### 2.5 Interventions

**FIGURE 3:**
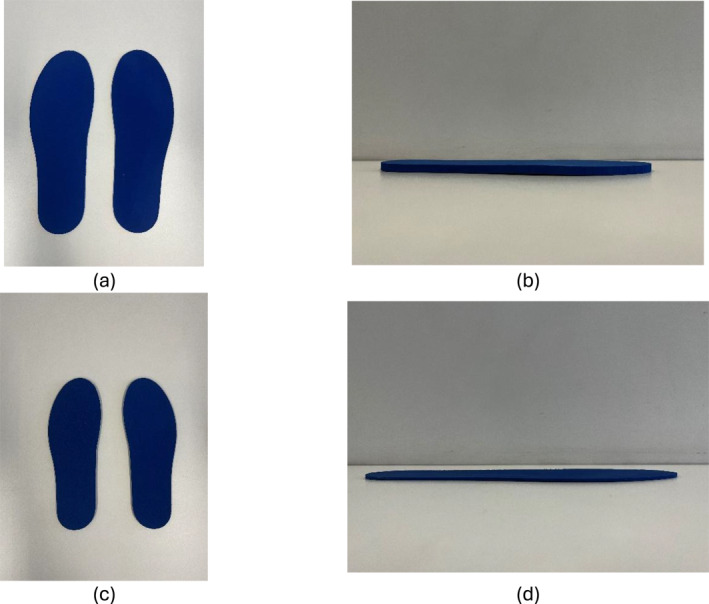
Cushioned insoles as viewed from above (a) and the side (b) and sham insoles as view from above (c) and the side (d).

Participants in both groups receive three pairs of insoles packaged identically and of similar appearance and colour (Figure 3). Insoles are trimmed and fitted to the participant's footwear by research staff in the laboratory. Participants unable to attend the laboratory are mailed the insoles and instructions for fitting them to their shoes. They are instructed to insert a pair of insoles whenever shoes are worn for 12 weeks, replacing the insoles with a new pair every 4 weeks. Participants in both groups are asked to avoid commencing new treatments specifically for the study foot during the 12‐week period, where possible.

#### 2.5.1 Cushioned Insoles (Intervention Group)

Three pairs of flat 6 mm blue EVA 120 (Shore A 20) single‐density, closed‐cell polyethylene foam insoles are provided to participants in this group. These low‐density, thicker insoles have been shown to reduce peak plantar pressures beneath the hallux [17].

#### 2.5.2 Sham Insoles (Control Group)

Three pairs of flat 2 mm blue EVA 450 (Shore A 65) single‐density, closed‐cell polyethylene foam insoles are provided to participants in this group. These were selected to match the appearance of the cushioned insoles but provide minimal cushioning (the active ‘ingredient’ of the intervention group).

To investigate the effect of the cushioned and sham insoles on plantar pressure, we recruited 10 adults for a small exploratory biomechanical study. All participants provided informed consent, and ethical approval was included in the wider trial application (see below). The results showed that mean and peak hallux plantar pressures were lower when walking in the 6 mm cushioned insole compared with the 2 mm sham insole (mean difference = −21.12 kpa and −76.36 kpa, respectively), while mean and peak hallux plantar pressures were slightly greater when walking in the sham insole compared with no insole (i.e., shoe only) (mean difference 12.33 and 20.46 kpa, respectively) (see Supporting Information S1: Table S2).

### 2.6 Outcome Measures

The schedule of enrolment, interventions and assessments is outlined in Table 1. Outcomes are measured at baseline and 12 weeks, unless indicated otherwise. If a participant does not complete their follow up questionnaire by the due date, automated reminders are sent every 4 days until 16 days after the due date. They are also phoned by a study researcher around day 5–7. If the participant has still not completed their questionnaire 16 days after the due date, they are considered lost to follow up and this is documented.

**TABLE 1:** Schedule of enrolment, interventions and assessments.

Timepoint	Study period				
	Enrolment	Allocation	Post‐allocation		Close‐out
	−*t*1	0 w	4 w	8 w	12 w
Enrolment					
Eligibility screen	X				
Informed consent	X				
Allocation		X			
Interventions					
Cushioned insoles		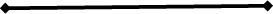			
Sham insoles		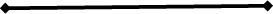			
Assessments					
Primary outcome					
Severity of first MTP joint walking pain (11‐point NRS)		X			X
Secondary outcomes					
Foot health status questionnaire pain subscale		X			X
Foot health status questionnaire physical function subscale		X			X
Severity of first MTP joint pain overall		X			X
Achievement of a global improvement in pain					X
Achievement of a global improvement in function					X
Quality of life (assessment of quality of life II instrument)		X			X
Physical activity scale for the elderly		X			X
Brief fear of movement scale for osteoarthritis		X			X
Other outcomes					
Use of other OA treatments		X			X
Adverse events					X
Pain at other sites		X			X
Foot pain location		X			X
Self‐rated adherence to wearing shoes					X
Percentage of time wearing the study insoles when wearing shoes per day			X	X	X
Number of participants who stopped wearing the study shoes					X
Descriptive measures		X			
Co‐morbidities		X			
Duration of symptoms		X			
First MTP joint radiographic disease severity		X			
Hallux valgus severity		X			
First MTP joint range of motion^a^		X			
Current employment status		X			
Treatment expectation		X			
Credibility/expectancy		X			
Foot posture index^a^		X			
In‐shoe plantar pressure^a^		X			

Abbreviations: MTP = metatarsophalangeal, NRS = numerical rating scale.

^a^ Only collected in participants who attend the laboratory.

#### 2.6.1 Primary Outcome

The primary outcome is the 12‐week change in average pain severity in the first MTP joint during walking over the last week, assessed using an 11‐point NRS (0 = no pain; 10 = worst possible pain). This outcome has an MCID of 1.8 points [27], and is recommended for use in OA RCTs [28, 29].

#### 2.6.2 Secondary Outcomes

##### 2.6.2.1 Change in the Foot Health Status Questionnaire (FHSQ) Pain Subscale

The FHSQ pain subscale comprises four items assessing foot pain [30]. Responses for each item are rated on a 5‐point Likert scale from 1 (not at all) to 5 (extremely), and are recoded to provide a score between 0 (worst foot health) to 100 (best foot health).

##### 2.6.2.2 Change in the Foot Health Status Questionnaire (FHSQ) Function Subscale

The FHSQ physical function subscale contains four items that assess the extent to which foot pain interferes with daily functional activities [30]. Responses for each item are rated on a 5‐point Likert response from 1 (not at all) to 5 (extremely), and are recoded to provide a score between 0 (worst foot health) and 100 (best foot health).

##### 2.6.2.3 Change in the Severity of First MTP Joint Pain Overall

Overall first MTP joint pain is rated using an 11‐point NRS for average overall pain over the past week (i.e., during weightbearing and non‐weighting). The scale ranges from 0 to 10, where 0 = no pain and 10 = worst pain imaginable.

##### 2.6.2.4 Achievement of Global Improvement in Pain and Function

Global improvement in (i) pain and (ii) function since baseline is assessed at 12 weeks using a seven‐point global rating of change scale, with response options from ‘much worse’ to ‘much better’. Participants who report being ‘moderately better’ or ‘much better’ are classified as improved; all other responses are classified as not improved.

##### 2.6.2.5 Change in Quality of Life (AQoL‐6D)

Quality of life is measured using the 20‐item Assessment of Quality of Life II (AQoL‐6D) scale, which covers topics including Independent Living, Relationships, Mental Health, Coping, Pain and Senses [31]. Total scores range from −0.04 to 1.00, with higher values indicating better quality of life.

##### 2.6.2.6 Change in the Physical Activity Scale for the Elderly (PASE) Score

Physical activity is self‐reported using the PASE, which assesses occupational, household and leisure activities over the previous week [32]. Total scores range from 0 to 400+, with higher scores indicating greater activity.

##### 2.6.2.7 Change in the Brief Fear of Movement Scale for Osteoarthritis

The Brief Fear of Movement Scale for Osteoarthritis comprises six statements assessing fear of injury or re‐injury from movement [33]. Each item is rated on a four‐point Likert scale from 1 = ‘strongly disagree’ to 4 = ’strongly agree’. Total scores range from 6 (minimal fear) to 24 (maximal fear), with higher scores indicating greater fear of movement.

#### 2.6.3 Other Outcomes

##### 2.6.3.1 Use of OA Treatments

Participants complete a custom table indicating the frequency of use of a range of pain and OA medications, supplements, and co‐interventions over the previous 12 weeks. Participants reporting use of a drug or supplement at least once per week in the past month are classified as current users of that medication or supplement. Participants reporting use of a co‐intervention at least once in the past 12 weeks are classified as current users of that co‐intervention. We will report the number and proportion of participants who are current users of OA treatments.

##### 2.6.3.2 Adverse Events

Adverse events are defined as any new problem affecting the study foot or elsewhere that the participant attributes to the insoles or participation in the trial AND that meets at least one of the following: (i) caused increased pain and/or interfered with function for 2 days or more, and/or (ii) prompted the participant to seek treatment from a health professional. Adverse events are self‐reported at 12 weeks using a customised table. We will report the proportion of participants experiencing adverse events, the types of events, and the number of participants who discontinue the study because of adverse events. Serious related adverse events are unlikely due to the nature of the intervention.

##### 2.6.3.3 Pain at Other Sites (Hips, Knees, Ankles, Other Foot Sites, Spine)

Pain at other sites will be recorded as (1) the number of other pain sites reported, and (2) pain severity, assessed using an 11‐point NRS for average pain in the past week for each site. We will report the number of pain sites and NRS scores for each site.

##### 2.6.3.4 Foot Pain Location

Participants indicate foot pain location by selecting a numbered region on a foot manikin. We will report the proportion of participants reporting pain at each foot location at baseline.

##### 2.6.3.5 Self‐Rated Adherence With Wearing Insoles Over 12 Weeks

Overall adherence to wearing the insoles over the 12‐week period is rated by participants at 12 weeks on an 11‐point NRS, where 0 indicates insoles not worn at all when wearing shoes, and 10 indicates insoles worn completely as instructed when wearing shoes. Scores range from 0 to 10, with higher scores indicating better adherence.

##### 2.6.3.6 Percentage of Time Wearing the Study Insoles When Wearing Shoes Per Day

Participants record insole and shoe wear daily in a logbook over the prior week (1 week snapshot) at weeks 4, 8 and 12. We will calculate the percentage of hours during which study insoles were worn when wearing shoes, averaged over the recording week. We will report the number and proportion of participants classified as adherent (average daily insole wear ≥ 70% of time when shoes were worn) and non‐adherent (average daily insole wear < 70% of time when shoes were worn) at each of weeks 4, 8 and 12, and for the entire 12‐week intervention period.

##### 2.6.3.7 Number of Participants Who Stopped Wearing the Study Insoles

Participants will indicate if they stopped wearing the study insoles at any time during the study (Yes/No) at 12 weeks. Those reporting ‘Yes’ will indicate when and why they ceased wearing the insoles; these reasons and timing will be reported descriptively.

##### 2.6.3.8 In‐Shoe Foot Plantar Pressures

Participants attending the laboratory will be asked to walk across an 8 m walkway at their usual pace for three trials in their usual shoes at baseline. Pedar system insoles will be placed inside the shoes, and participants will complete testing with and without their allocated insole, in random order. Pressure (kPa) and force (N) under the hallux will be extracted and used for moderator analyses (see below).

#### 2.6.4 Descriptive Measures

Descriptive measures are assessed at baseline, and include height, weight, body mass index, age, sex, gender, comorbidities (recorded using the Self‐Administered Comorbidity Questionnaire [34]), duration of symptoms, radiographic severity (osteophyte and joint space narrowing scores, assessed using a validated atlas [22]), hallux valgus angle (measured in degrees from x‐rays using imaging software), first MTP joint range of motion (measured in degrees using a goniometer; only for those attending the laboratory), employment status, treatment expectation (assessed on a five‐point ordinal scale from ‘no effect at all’ to ‘complete recovery’), credibility and expectancy (assessed using the Credibility/Expectancy Questionnaire [35] following insole allocation), and foot posture (assessed using the foot posture index [36]; only for those attending the laboratory).

### 2.7 Statistical Analysis

All statistical analyses will be performed by study biostatisticians while blinded to group allocation and will follow a prespecified statistical analysis plan finalised prior to analyses. Analyses will include all participants according to their randomised allocation. Demographic and baseline participant characteristics will be summarised as appropriate using means and standard deviations for approximately symmetrically distributed continuous variables, medians and interquartile ranges for other continuous variables, and counts and percentages for categorical variables. The primary outcome will be compared between groups using linear regression adjusted for baseline pain. The primary hypothesis will be evaluated by estimating the between‐group difference in mean change in pain from baseline to 12 weeks, a two‐sided 95% confidence interval and p value. If greater than 5% of primary outcome data are missing, multiple imputation will be used under a missing at random assumption. Continuous secondary outcomes will be analysed similarly to the primary outcome. For binary secondary outcomes, groups will be compared using risk ratios and risk differences, estimated using log‐binomial regression models. Similar analyses will be conducted for adverse events. A supplementary analysis estimating treatment effects assuming full adherence (calculated from insole and shoe wear, recorded daily in a log book for a 1 week snapshot every month, and wearing insoles ≥ 70% of the time when shoes were worn) will be conducted using an instrumental variables approach [37]. Standard diagnostic plots will be used to check model assumptions.

To assess the effect of prespecified modifiers of (i) body mass index (BMI), (ii) first MTP joint range of motion (only for those attending the laboratory), and (iii) peak hallux plantar pressure (only for those attending the laboratory) on the primary outcome of first MTP joint walking pain, interaction terms between randomised group and each moderator will be included in separate models. Hypotheses and rationale for moderators is described in Table 2. Additional supplementary and sensitivity analyses may be conducted and will be prespecified in the statistical analysis plan.

**TABLE 2:** Study moderators, hypothesised effect and proposed rationale.

Moderator	Hypothesis	Rationale
Body mass index (BMI)	We hypothesise that either a higher or lower BMI may moderate pain reductions with cushioned insoles (relative to sham insoles).	Pain reductions in those with a higher BMI may be lower based on the likelihood that a greater mass will compress the cushioning properties of the cushioned insoles, rendering them less capable of leading to symptomatic benefits. Alternatively, pain reductions in those with a higher BMI may be greater given that those with a higher BMI will have higher loads in the hallux and first MTP joint and therefore may have more potential to benefit from the cushioned insoles.
First metatarsophalangeal (MTP) joint range of motion	We hypothesise that pain reduction with the cushioned insoles (relative to sham insoles) will be greater in those with less range of motion compared to those with more range of motion.	Reduced first MTP joint range of motion increases first MTP joint loading, and therefore these participants may have a greater propensity for pain reduction.
Peak hallux plantar pressure	We hypothesise that pain reduction with the cushioned insoles (relative to sham insoles) will be greater in those with higher hallux plantar pressures compared to those with lower hallux plantar pressure.	People with higher hallux plantar pressures have a greater scope for pressure reductions with cushioned insoles, and thus greater potential for pain reduction.

### 2.8 Sample Size Calculation

A sample of 108 participants (54 per arm) provides 90% power to detect a between‐group MCID of 1.8 NRS points [27] in walking pain at 12 weeks, with a two‐sided alpha of 0.05. This calculation assumes a standard deviation of 2.6 for change scores (equal across groups) and a baseline–follow‐up correlation of 0.25 based on our previous RCT [21], and allows for 20% attrition.

### 2.9 Patient and Public Involvement

We recruited five consumers aged over 45 years with first MTP joint pain to help us refine our outcome measures and to provide feedback on the insoles. Consumers attended our laboratory where they completed our study outcomes and gave feedback on comprehension and ease of use. They then wore the insoles for the subsequent 4 weeks, after which they provided feedback regarding their likelihood of wearing the study insoles, as well as their comfort and acceptability.

## 3 Ethics and Dissemination

The trial will be conducted in accordance with the Declaration of Helsinki. Informed consent is obtained from all participants prior to enrolment. This study has been approved by the University of Melbourne Greater than Low Risk Human Research Ethics Committee (reference: 2023‐26451‐41783‐4). Results will be disseminated via peer‐reviewed journals, scientific conferences, information on our website and lay summaries for participants.

## 4 Discussion

The SOLE RCT will provide the first clinical trial evidence on whether cushioned insoles reduce walking pain and improve other symptoms more than flat sham insoles in people with symptomatic radiographic first MTP joint OA. Given the limited non‐surgical treatment options for the condition, and the biomechanical rationale that cushioning reduces hallux plantar pressures [17], cushioned insoles offer a plausible, promising and untested treatment approach. Cushioned insoles were not identified as a management approach in an international survey of podiatrists and physiotherapists [38], or in an analysis of a national general practitioner database [39], suggesting they could provide a new treatment option that is not currently used in routine clinical practice. If they are found to provide clinically meaningful benefits, they would offer a low‐cost, low‐burden management strategy readily implementable in clinical practice.

A novel aspect of the SOLE RCT is that we provide the option to mail study insoles to participants who cannot attend our laboratory in person. This enables the inclusion of participants from remote and/or regional locations who otherwise would not have been able to participate in our trial. Whilst this is a strength of our study, this approach relies on participants providing their correct shoe size, and fitting the insoles themselves. Thus, there is potential for the insoles to not fit these participant's shoes correctly, which could adversely affect insole comfort and their therapeutic effect. Another potential limitation of our trial is that we are not assessing whether participants change insoles at weeks 4 and 8 as instructed.

Clinical guidelines exist for the management of OA of the knee and hip, but there are no guidelines to advise on the evidence‐based management of OA at the first MTP joint. Findings from the SOLE RCT will help to build the limited RCT evidence base for this condition, and will help inform future clinical guidelines for the management of first MTP joint OA.

## Author Contributions

**Kade L. Paterson:** conceptualization, investigation, methodology, project administration, supervision, writing – original draft preparation. **Kim L. Bennell:** resources, writing – review and editing. **Adam Bryant:** writing – review and editing. **Peixuan Li:** data curation, formal analysis, writing – review and editing. **Anurika P. De Silva:** data curation, formal analysis, writing – review and editing. **Sam Shearer:** investigation, project administration, writing – review and editing. **Rana S. Hinman:** conceptualization, investigation, methodology, writing – review and editing.

## Funding

The trial was partially funded by KLP's National Health & Medical Research Council Emerging Leadership Investigator Grant (#1174229). RSH is supported by a National Health & Medical Research Council Leadership Investigator Grant (#2025733). KLB is supported by a National Health & Medical Research Council Leadership Investigator Grant (#1174431). The funders have no role in conduct, analysis or reporting of this study.

## Conflicts of Interest

The authors declare no conflicts of interest.

## Supporting information


Supporting Information S1

## Data Availability

Data sharing not applicable to this article as no datasets were generated or analysed during the current study.
